# Searching for effective preprocessing method and CNN based architecture with efficient channel attention on speech emotion recognition

**DOI:** 10.1038/s41598-025-19887-7

**Published:** 2025-09-24

**Authors:** Byunggun Kim, Younghun Kwon

**Affiliations:** 1 Department of Applied Artificial Intelligence, Hanyang University(ERICA), Ansan, 425-791 Kyunggi-Do Republic of Korea; 2 Department of Applied Physics, Hanyang University(ERICA), Ansan, 425-791 Kyunggi-Do Republic of Korea

**Keywords:** Speech emotion recognition, Convolutional neural network, Efficient channel attention, Log-Mel spectrogram, Data augmentation, Engineering, Mathematics and computing

## Abstract

Recently, Speech emotion recognition (SER) performance has steadily increased as multiple deep learning architectures have adapted. Especially, convolutional neural network (CNN) models with spectrogram data preprocessing are the most popular approach in the SER. However, designing an effective and efficient preprocessing method and a CNN-based model for SER is still ambiguous. Therefore, it needs to search for more concrete preprocessing methods and a CNN-based model for SER. First, to search for a proper frequency-time resolution for SER, we prepare eight different datasets with preprocessing settings. Furthermore, to compensate for the lack of emotional feature resolution, we propose multiple short-term Fourier transform (STFT) preprocessing data augmentation that augments trainable data with all different sizes of windows. Next, because CNN’s channel filters are core to detecting hidden input features, we focus on the channel filters’ effectiveness on SER. To do so, we design several types of architecture that contain a 6-layer CNN model. Also, with efficient channel attention (ECA) that is well known to improve channel feature representation with only a few parameters, we find that it can more efficiently train the channel filters for SER. With two different SER datasets (Interactive Emotional Dyadic Motion Capture, Berlin Emotional Speech Database), increasing the frequency resolution in preprocessing emotional speech can improve emotion recognition performance. Consequently, the CNN-based model with only two ECA blocks can exceed the performance of previous SER models. Especially, with STFT data augmentation, our proposed model achieves the highest performance on SER.

## Introduction

Emotion recognition can help in more natural interaction between computers and humans by understanding emotions, which are unique properties that only humans have^1^. To this end, research has been actively conducted recently to understand emotions using various data^2,3^. Among them, Speech emotion recognition (SER) is the technique in which a computer can recognize the inherent emotional features of a human’s speech signal^4^. In particular, interest in the human-computer interaction (HCI) systems has arisen^5^, and SER has noticed that some applications, such as psychotherapy^6^ and consultation calls^7^, require emotional labor. However, it is hard to understand how human speech emotions can be represented in terms of the exact values using common standards. This is because each person’s method of recognizing emotions differs depending on their personality and culture^8^. The ambiguity of human emotions is one of the main challenges in developing an accurate SER model^9^.

Therefore, many studies have attempted to use the deep learning-based models^10–14^, that can be trained directly from the emotional speech data. In particular, deep learning models have been shown to greatly outperform existing machine learning-based models. However, many studies have tried to solve the SER problem with their own preprocessing methods and model structures. As a result, there is still ambiguity on how to set input data and models for SER. Therefore, we want to find clues about more clearly defined preprocessing settings and model design for SER.

First, for the speech preprocessing method, we choose a short-term Fourier transform (STFT)-based preprocessing. STFT can obtain rich speech features by obtaining series of frequency features with a short time window^15^. However, there is a resolution trade-off between time and frequency due to the short duration window. In particular, in the case of SER, it is necessary to learn more detailed paralinguistic expressions than linguistic features^16^. So, the resolution problem can be more critical in SER^17,18^. For this reason, STFT preprocessing settings suitable for SER are essential.

Next, recently, the convolutional neural network (CNN) based models trained with speech spectral features are proposed^19–25^. A CNN-based model has two advantages. First, owing to the convolutional layer’s weight sharing, a relatively small number of trainable parameters can be used. Second, a deep CNN layer model makes it possible to learn global context features using filters of only a small size^26–28^. Therefore, the CNN model, which can learn the context of emotional speech utterances, performs better than the other structures.

However, to learn the overall context of the input data with a CNN-based model, a sufficient number of deep layers must be stacked, or a larger filter kernel must be used. Therefore, attention modules have been proposed to cover the CNN layer’s weakness for the SER^29–34^. Xu et al.^31^ proposed a multiscale area attention, which applies the transformer-type attention mechanism^35^ to the CNN-based model. This significantly improves the recognition performance by dividing the time-frequency spatial information into granular perspectives. Guo et al.^33^ proposed spectral temporal channel attention, which is a modified version of bottleneck attention module (BAM)^36–38^. Therefore, it not only focuses on spatial features but also attention to channel features. In addition, it has an independent attention learning structure in all the axes of the input features. However, channel attention requires more learning parameters than spatial attention because of the two multi-layer perceptron (MLP) layers. More trainable parameters are required when examining the attention structure, it causes overfitting problems when trainable samples are leaked, such as in SER^39,40^. In particular, this problem becomes more severe as the dimension of the data to be learned increases, so a more efficient attention mechanism structure is required^41^.

Therefore, in this study, we search for an efficient attention structure that can improve emotional feature expression with only a few learning parameters while maintaining a deep CNN-based model by focusing on CNN’s filter training. CNN’s filter is a core component in CNN-based models to extract not only spatial complex structures but also the relation of each local feature^42^. Furthermore, when the filters are more effectively used, the CNN-based model can achieve more computational efficiency^43,44^. Through experiments, we observe that CNN’s channel features are essential for emotion classification performance. Therefore, to focus on the important channel feature, we adopt the efficient channel attention (ECA)^45^. Because the ECA mainly uses a 1-D convolution layer, it is highly efficient because it requires only a few trainable parameters equal to the kernel size^46^. For the first time, we explore how to effectively use the ECA on the SER problem.

**Fig. 1:**
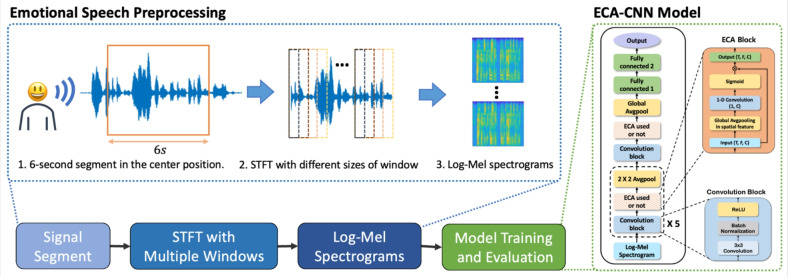
The overall pipeline of speech emotion recognition with CNN-based efficient channel attention architectures.

We also search for a more appropriate preprocessing method to better represent the emotional features. Previous studies have preprocessed emotional speech signals using different methods; therefore, it is difficult to compare the results. Therefore, we prepare the eight different types of preprocessed datasets with the interactive emotional dyadic motion capture (IEMOCAP) corpus^47^ and Berlin Emotional Speech Database (EMODB)^48^. Specifically, we choose the log-Mel spectrogram preprocessing method first. We preprocess the speech signals with different and overlapping window sizes using short-term Fourier transformation (STFT). As a result, we can observe that a preprocessing method with a large window size can accurately represent the emotional features. Also, to deal with less emotional speech data in the SER domain, we propose STFT data augmentation. With the number of different settings of overlapping window sizes, STFT data augmentation simply overcomes the frequency-time resolution problem to extract emotional features. Figure 1 shows the overall pipeline used in the experiments.

In summary, our contributions to this paper are below:

- With the experiments, we showed that a CNN’s channel complexity significantly affects the SER performance. Therefore, the ECA was applied to the SER domain for the first time. As a result, the ECA efficiently improved the emotion classification results with only a few training parameters. In particular, ECA is effective in the deep layer of the CNN model, which has many channels instead of not being used in all CNN layers like the original method.
- We conducted experiments with eight datasets preprocessed under various STFT settings to search for the effective preprocessing method for expressing emotional features. The precision of our results proved that representing a relatively high-frequency resolution is significantly effective in emotion classification.
- In the field of SER, the need to learn data poses a significant challenge. To address this, we proposed STFT data augmentation, which uses various preprocessed settings in STFT to supplement the expression of emotional features. STFT data augmentation had a profound impact, resulting in a substantial enhancement in emotion classification performance. When using a CNN-based model with ECA applied, we achieved the highest performance to date. (IEMOCAP: 80.28UA 80.46WA 80.37ACC, EMODB: 87.67UA 88.04WA 87.85ACC)

This paper’s overall composition is as follows: Section “Related works” describes several CNN-based models with attention modules proposed for SER. Section “Proposed method” introduces our proposed method. Section “Experimental results” presents the details of the experimental settings and evaluation results when the ECA is applied to our CNN-based model. Finally, Section “Conclusion” concludes the paper.

## Related works

Many different attention methods have been proposed. In this section, we present an overview of the development flow of CNN-based models using several attention methods. We divide the contents whether the recurrent neural network (RNN) is used or not in the CNN-based model.

### CNN-RNN models with attention mechanism

The CNN can effectively learn local spatial features from the data. It is also possible to learn the global spatial features, such as the context of the data, when stacking multiple CNNs. However, if the model stacks more layers, its complexity increases. In the SER problem, increasing the model complexity is critical. Therefore, to train the emotional context from the speech signals while sustaining the model complexity, most studies have proposed a combination model of a CNN and RNN^19–21^. Although it is possible to learn the temporal features of speech using RNN, there are limitations to learning long sequences. Therefore, to compensate for the limitations, many models that combine attention mechanisms with RNN have been proposed.

M. Chen et al.^22^ proposed a CNN-LSTM-Attention model to aggregate the hidden states in each time step. This enables effective learning even for long sequences. In addition, for more comprehensive context learning, ADRNN^23^, which uses residual connections and dilated convolution layers together, and ASRNN^24^, which compensates for the shortcomings of RNN through a sliding RNN method, have been proposed.

However, models using CNN-RNN-Attention layers forcefully learn the spatio-temporal features together. However, models consisting of CNN and RNN models in parallel are proposed to independently learn spatio-temporal features^49^. Zhao et al.^50^ proposed a structure that separates LSTM and CNN in parallel and applies independent attention. Furthermore, Z. Chen et al.^34^ proposed AMSnet, which is a parallel model that effectively synthesizes features through a connection attention mechanism.

### CNN-based models with attention mechanism

Recently, there has been a trend to use only CNN and attention layers without an RNN. Because RNN requires many more computational and training parameters than others, they focus on developing attention mechanism methods that help learn the context of the spatial features of the speech spectrogram. Xu et al.^29^ demonstrated effective emotional feature learning only through self-attention after the CNN layer for the spatial context learning.

Another attention mechanism method was proposed to enhance the feature learning of the CNN layers. Li et al.^30^ demonstrated the importance of frequency features that use frequential attention, in addition to spatial attention. Xu et al.^32^ proposed the ATDA, which applied independent self-attention to all feature axes (temporal, frequential, and channel) to compensate for the weakness of temporal feature learning owing to the lack of an RNN. Guo et al.^33^ proposed STC, which is a more efficient attention method for all feature axes of the CNN structure.

Furthermore, with the era of transformer^35^ in the deep learning model, Saleem et al.^51^ and Li et al.^52^ try to adopt a transformer-based attention mechanism for the SER. Saleem et al.^51^ proposed DeepCNN, which contains the parallel CNN model with a convolutional transformer. Oppositely, Li et al.^52^ use only a transformer-based model to extract the relationship in the Mel-spectrogram without the CNN model

We further explore this trend and attempt to find an attention structure that is efficient and effective in learning emotional features while using a deep-layer CNN structure. In particular, for efficient attention, we focused on learning the channel features that contained the context information of the input data in the CNN layer. Therefore, we applied the ECA module to the SER problem and obtained improved emotion classification performance than before.

## Proposed method

In this section, we explain how to find the effective and efficient preprocessing method for the CNN-based model architecture with the ECA block for SER. For this purpose, we use several types of STFT preprocessing settings, which are frequently used for previous speech emotion recognition studies.

**Fig. 2:**
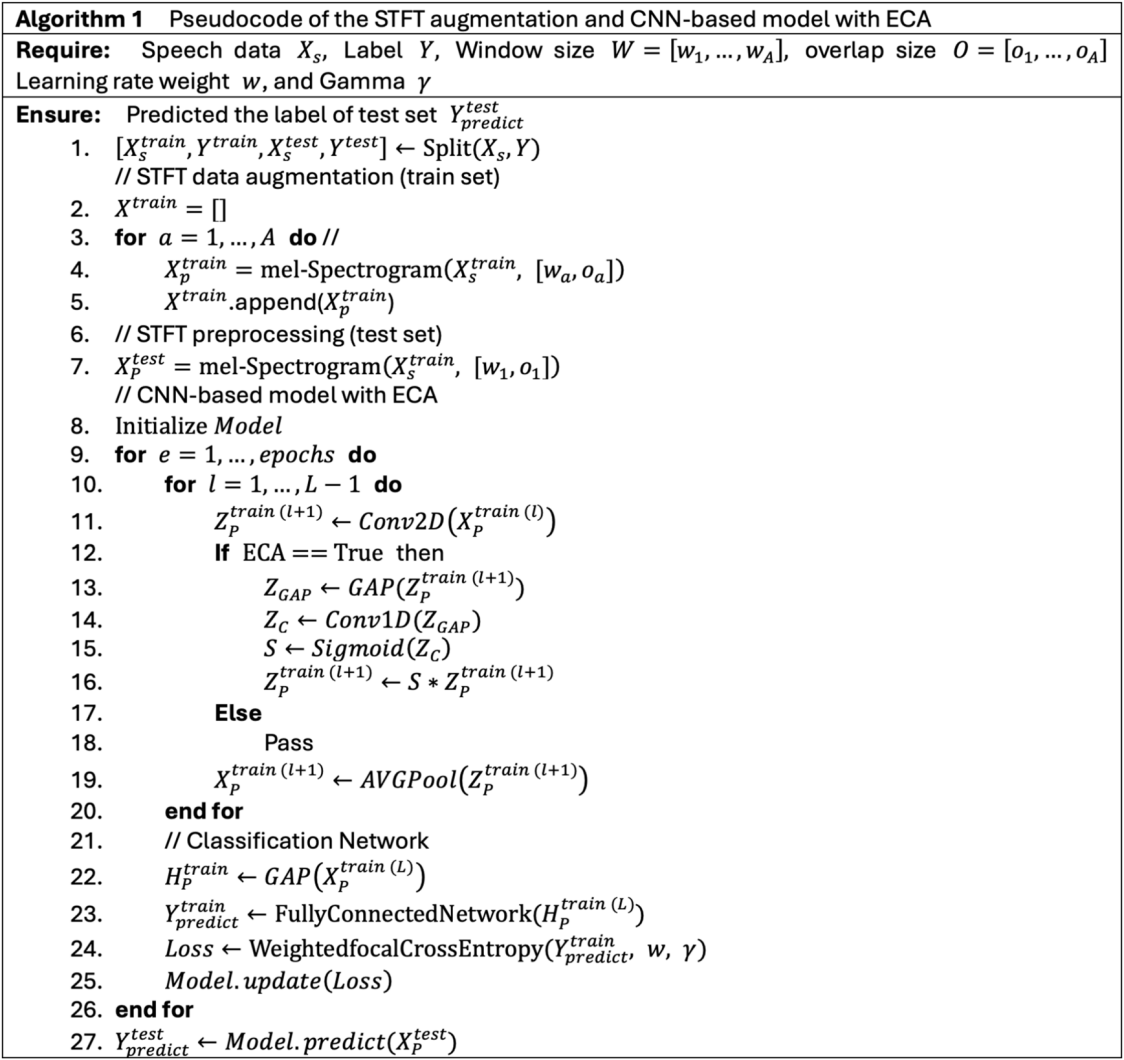
The pseudocode of the proposed method.

Also, we search for a CNN-based model architecture with the ECA block for SER. As a result, It shows that properly selecting channel size and number of ECA blocks can effectively elevate the representation of the channel features. The detailed pseudocode of the proposed method can be found in Fig. 2.

### STFT and data augmentation

The STFT is a method used to obtain the spectrum of the frequency features by dividing a signal into short periods. So, it can represent paralinguistic features in human speech. For that reason, STFT is widely used in emotional speech processing. However, in the SER domain, how to set the STFT to get a spectrum has yet to be discovered. Therefore, we want to search for the clue to extract richer emotional features with STFT. The STFT can be described as follows:

1 $$\begin{aligned} X_t(f)=\sum _{\tau =-\infty }^{\infty } x(\tau )w(\tau - ts)e^{2\pi i f \tau } \end{aligned}$$

2 $$\begin{aligned} s = l - o \end{aligned}$$

The output $X_t(f)$ is obtained by applying a windowing function (*w*) to a signal $x(\tau )$ in an interval of the windowing function. The output $X_t (f)$ then becomes a feature of the frequency. The windowing function moves according to stride (*s*), which is determined by the windowing length (*l*) and the overlapping length (*o*).

Although the STFT can show simultaneously the time-frequency feature, it has a time-frequency resolution trade-off^15^. Whether we set the window length long or short, the information in the spectrum can change. If the window length is longer, the frequency resolution increases; however, the resolution in time decreases. To do so, we have two questions: (1) Which information is more important for SER? (2) How can we train the model with richer input resolution in frequency instead of time?

With question (1), we search for the proper window size to represent emotional features. For this purpose, we prepare datasets with eight different STFT settings. The details of the STFT setting can be found in Table 1. To list these settings, we reference the window size of the previous studies (from 16 ms to 50 ms). And, to normalize the input size of the spectrum, we adjust the overlap size. More details can be found in the Subsection “Data preprocessing”.

In SER, the results from question (1) show that the frequency feature is more important than the time feature. For that reason, with question (2), we proposed the STFT data augmentation to enhance the diversity of frequency features. As a kind of multi-resolution method^17,18^, STFT data augmentation preprocesses the raw data with different window sizes to complement the time-frequency resolution trade-off. So, it can extract much richer emotional features from speech data. The experiment results show that with only STFT augmentation, the emotional recognition highly improved.

### CNN-based architecture

In the CNN-based model, an appropriate number of weight filters and kernel sizes is important to get better performance. Especially, we mainly focus on some weight filters called “channel size”. As the core component of the convolution layer, the channel size is a hyperparameter related to the information capacity that captures the spatial complex features from data. If we use many weight filters, spatial information can be sensitively extracted from the input data. However, this leads to a more robust overfitting of the training dataset. For that reason, the proper number of channels can lead the model to more efficiently and effectively^42–44^.

**Fig. 3:**
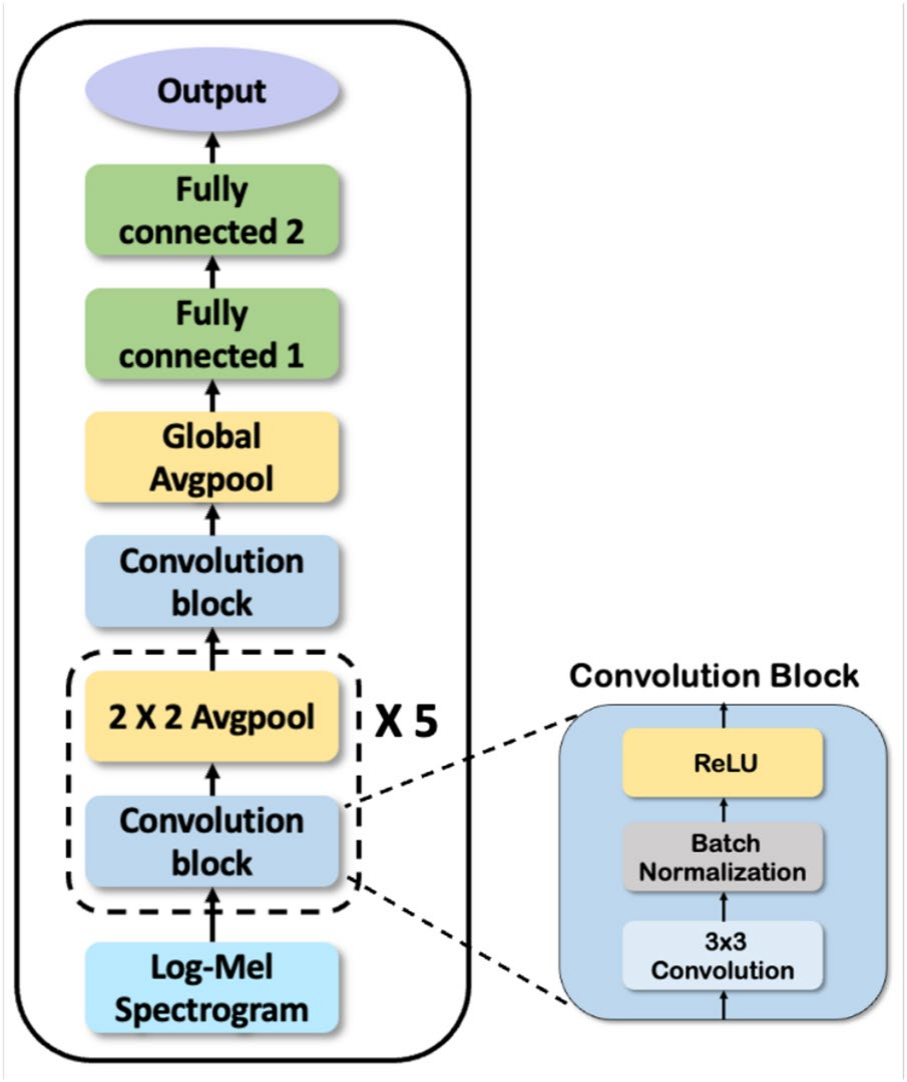
CNN-based model architecture. It consists of six convolution blocks and two fully connected layers.

To do so, we design a 6-layer CNN architecture to determine an adequate channel size for speech emotion recognition. The structure design of the CNN-based model was based on a previously proposed model for SER. Figure 3 shows the overall architecture of the CNN-based model. It is composed of six Convolutional blocks and a pooling layer. And, two fully connected layers are used for emotion classification.

The detailed model structure is as follows. First, we chose a convolution block, which is commonly used in image classification models. The convolution block consists of three layers: convolution layers with $(3 \times 3)$ kernel size, batch normalization, and ReLU activation. To find the adequate channel size in each convolution layer, we selected the initial channel size in multiples of 16. We multiplied by a scaling factor (*n*) to scale up the channel size.

Next, we used the average pooling layer after all the convolution blocks. It can efficiently subsample the hidden features without vanishing the features. Exceptionally, in the sixth pooling layer, we used global average pooling to connect with the next layer. Finally, two fully connected layers were used in the classification layer. The weight of each fully connected layer was set to be the same as the output channel size of the last convolution layer. The details of the parameter settings of the CNN-based model are listed in Table 3.

### ECA module for SER

In the convolution layer, channel filters can extract the local temporal and frequential features of emotional speech. However, because the SER domain has a smaller number of trainable emotional speech samples than other domains, it has the limitation of enhancing the emotional representation of the filters by only searching for the proper number of filters. For that reason, we modified and adopted the original ECA^45^ into the CNN-based model, which can effectively and efficiently improve the channel filter’s rule locally.

The ECA is a type of layer that applies a self-attention mechanism^35^. The self-attention mechanism comprises three components: query (*Q*), key (*K*), and value (*V*). These components are used to attend to the crucial information from input features. Each component’s role is as follows. A query is a “question” about what is essential in the input features. The key is “hint,” which uses how similar the query is. This helps to find the most appropriate information in the input features. The value is the “real answer” that exists in pairs with keys and is used as the output features of self-attention.

3 $$\begin{aligned} \textrm{Attention} (Q, K, V) = \textrm{Softmax}\left( \frac{{QK}}{ \sqrt{n}} \right) V \end{aligned}$$

The most common self-attention mechanism in (3) is the inner-product attention. In the first step, each query is multiplied by the multiple keys to obtain the relevant score matrix. In the second step, the score matrix is represented by a probability distribution within each query using the Softmax function. In the final step, the output is represented by a linear combination of all the values with a probability distribution. In summary, the output is the weighted value obtained that is most closely related to the query indirectly through the keys.

4 $$\begin{aligned} \textrm{ECA} (Q, V) = \sigma (\mathrm {1DConv(Q)}) \otimes V \end{aligned}$$

5 $$\begin{aligned} Q = \textrm{GAP}(X) \end{aligned}$$

**Fig. 4:**
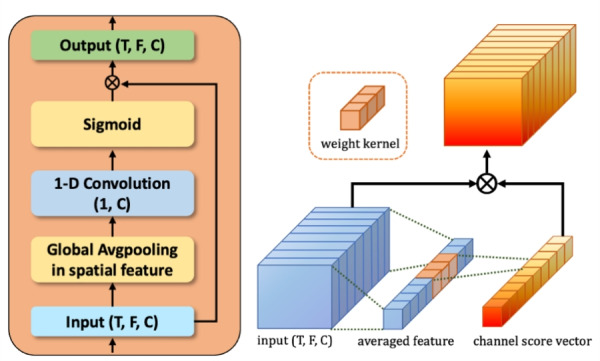
The design of an efficient channel attention module for SER.

To simplify the self-attention mechanism, the ECA omits the key from the existing self-attention structure. So, the importance of the value is judged by the score learned from the query itself. Also, without referencing the relation within all channel filters, it focuses on the local sparse relation of filters. Therefore, it leads to being more efficient with the CNN-based model. Equations (4), (5), and Fig. 4 show the progress of the ECA that we used for SER.

Specifically, first, the query represents the channel features in each input. With global average pooling, it contains temporal-frequency features in each channel without trainable parameters. Second, with the 1-D convolution layer, we trained the channel features’ relation efficiently. At last, with the sigmoid function, it returns each channel’s score from 0 to 1.

**Fig. 5:**
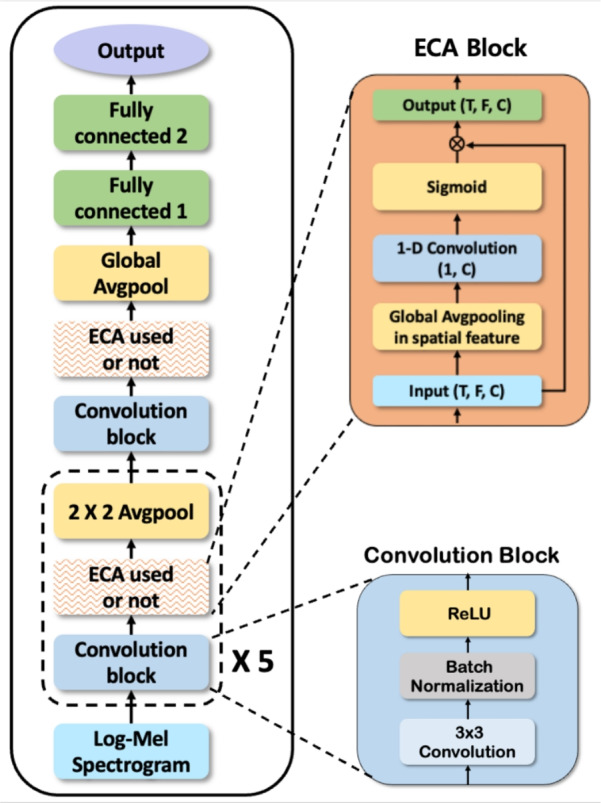
The ECA block position in the CNN-based model.

The original ECA and our ECA have some differences. The first difference from the original ECA is the omission of batch normalization. This change is made because batch normalization tends to extract global features of speech data rather than individual emotional features. The second difference is that we didn’t use ECA after all the convolution blocks. Instead, we selectively use ECA in specific layers. Figure 5 shows the ECA block used after the convolution block. We searched for more appropriate ECA block settings for the CNN-based model. To achieve this, we compared the different experiments using several kernel sizes and block positions in the model. Consequently, unlike the original ECA method used in all layers after the convolution block, using some convolution blocks with many channel features can be effective for emotion recognition performance. As a result, using ECA in deeper convolution blocks containing many channel features is more effective for emotion recognition performance.

Also, the effectiveness of ECA can be increased when augmentation data are used for training. Typically, sufficient data are required to use an attention-structured neural network effectively. Therefore, we used eight different versions of datasets as the augmentation data, as listed in Table 1. Consequently, the performance difference between the model combined with the ECA and the model without the ECA is visible.

### Weighted focal loss

Most speech emotion datasets have an unbalanced distribution, depending on the emotion labels. Therefore, when we use cross-entropy as a loss function, the model can be focused on a relatively large number of specific emotion labels (neutral and happiness). In addition, emotion classification is complex, depending on the label. For example, “happiness” and “angry” are often misclassified. Therefore, we used weighted focal loss to deal with those specific situations. The weighted focal loss can be expressed as follows.

6 $$\begin{aligned} \textrm{Weighted Focal} (p_i, w_i) = - \sum _{i=1}^{n_{class}} w_i (1-p_i)^{\gamma } \log (p_i) \end{aligned}$$

It has two properties. First, the focal loss^53^ achieves flexible learning rates in different emotional classes $(n_{class})$. The focal loss function is a slightly more generalized function of the cross-entropy weighted by the predicted probability of each emotion class $(p_i)$. For emotion classes with low probability values, the loss increases. This causes relatively more learning in backward updates. Conversely, emotion classes with high probability values resulted in relatively less learning. The hyperparameter gamma ($\gamma$) controls how dramatically the loss value changes. In our experiment, we set $\gamma =1$.

Second, to avoid imbalanced learning owing to the number of emotion classes, the loss function is multiplied by the learning rate weight $(w_i)$ based on the number of labels in the training dataset. The learning rate weight was obtained as the reciprocal ratio of the number of data for each label.

## Experimental results

**Table 1:** preprocessing setting in each version of datasets.

Dataset Version	Window	Overlap
1	15 ms	5 ms
2	20 ms	10 ms
3	25 ms	15 ms
4	30 ms	20 ms
5	35 ms	25 ms
6	40 ms	30 ms
7	45 ms	35 ms
8	50 ms	40 ms

**Table 2:** STFT setting used in previous proposed SER models.

Model Name	Method	Window	Overlap
STC^33^	spectrogram	16 ms	8 ms
AMSNet^34^	spectrogram	50 ms	25 ms
Area Attention^31^	log-Mel	40 ms	10 ms
MHA^54^	log-Mel	46 ms	23 ms
HNSD^14^	log-Mel	25 ms	10 ms
ATDA^32^	MFCC	48 ms	24 ms
TIM^55^	MFCC	50 ms	38.5 ms

### Datasets

For the experiment, we selected the two most popular datasets, called IEMOCAP^47^ and EMODB^48^. First, In IEMOCAP, ten individual actors (five men and five women) recorded their voices, facial movements, and overall behavior for five sessions to understand human emotions in various situations. The IEMOCAP’s data are divided into an improvised set, which contains improvised acting, and a script, which is acted through dialogue. Three or more annotators manually classified the emotional speeches, and the final decision was made through a majority vote. Next, the EMODB is a German database of emotional utterances spoken by ten individual actors (five men and five women). For the generalization, all actors performed all emotions with the same verbal content.

The data samples that we used in the experiment are as follows. In IEMOCAP, we selected only the Improvised set, considering situations in which human emotions can appear naturally. Next, we choose five emotional classes corresponding to “angry”, “sadness”, “happiness”, “neutral”, and “excited”. These classes are commonly used in various experiments. Moreover, to compensate for the data sample’s number of “happiness”, “excited” is considered as “happiness:”. The experiments were conducted using 2943 speech data samples containing four emotional classes (angry:289, sadness:608, happiness:947, neutral:1099). Next, in EMODB, we choose seven emotional classes corresponding to “angry”, “boredom”, “anxiety”, “happiness”, “sadness”, “disgust”, and “neutral”. And, it contained 535 speech data samples (angry:127, boredom:81, anxiety:69, happiness:71, sadness:62, disgust: 46, neutral:79)

### Data preprocessing

To efficiently represent the input data, we extracted the log-Mel spectrogram features from the speech signal. The data preprocessing steps are as follows.

First, signal segmentation is performed to equalize the length of the input data. Specifically, when we set the segmentation length as 6*s*, the signal segmentation processes with two cases. If the speech data were shorter than 6*s*, zero padding was performed at the beginning and end of the signal with the same length for the signal position in the center. If the speech was longer than 6*s*, both the beginning and ends of the signal were cut to the same size to contain as long an utterance as possible.

The details of signal segmentation settings in each dataset are as follows. In the IEMOCAP dataset, the speech samples had an average length of 4.5 *s* and varied from short ($\sim 0.5s$) to long ($\sim 30s$) speech. Therefore, the lengths of the speech samples were consistent at 6*s*, which was slightly longer than the average. In the EMODB dataset, the speech samples had an average length of 2.5*s* and varied from short ($\sim 1.2s$) to long ($\sim 9s$) Based on this population, the lengths of the speech samples were consistent at 3*s*.

Next, we prepared the different versions of the datasets to search for more effective preprocessing settings with different window sizes and overlaps in the STFT. Therefore, an interval was set based on previous studies. As listed in Table 2, most previous studies set the window size from 16 ms to 50 ms. Based on this, we chose eight different window sizes at 5ms intervals within a slightly wider range of 15 ms to 50 ms. The overlap size was adjusted to obtain the same size of input data.

In the final step, log-Mel filters were applied to effectively decrease the input data size. Specifically, we use 64-number Mel filters to increase frequency resolution. The detailed settings for each dataset version of the dataset are listed in Table 1. All preprocessing steps were conducted using MATLAB.

### Experimental setup and evaluation

We use 5-fold cross-validation for the entire dataset to ensure general SER performance. Samples from all the sets were randomly selected. To evaluate the model’s performance, we used the unweighted average accuracy (UA) and weighted average accuracy (WA). The WA is the overall classification accuracy of the test dataset. And the UA is the average accuracy for each class of the emotion categories. Additionally, to compare the balanced performance of the models, we calculate the average accuracy of WA and UA, called ACC^31,32^.

The PyTorch framework^56^, a deep learning framework, was implemented in all the experiments in this study. The specific hyperparameters of the models were as follows: All weight parameters were initialized with He initialization^57^. For the model’s optimization, we use the Adam optimizer^58^ with $10^{-4}$ initial learning rates and $10^{-6}$ decay rates. The batch size was set to 36, and the focal loss parameter $\gamma$ was set to 1. And the models were trained for 100 epochs. Finally, for the experiments, we use NVIDIA RTX 2080 Ti (12GB) and Intel(R) Xeon(R) Gold 6230 CPU @ 2.10GHz

**Table 3:** Parameter settings of each layer in baseline model.

Layer	Kernel size	Stride	# of Filters or Parameters
Conv1	(3,3)	1	$16 * n$
Conv2	(3,3)	1	$32 * n$
Conv3	(3,3)	1	$48 * n$
Conv4	(3,3)	1	$64 * n$
Conv5	(3,3)	1	$80 * n$
Conv6	(3,3)	1	$96 * n$
Linear1	-	-	$(96*n, 96*n)$
Linear2	-	-	$(4, 96*n)$
Avgpool	(2,2)	2	-

### Searching the proper channel size for CNN-based model architecture

To extract emotional features from speech data more effectively, we first experimented with the number of channel sizes used in a CNN-based model. The channel size is the most critical hyperparameter in the convolution layer. Also, the proper channel size is crucial to efficiently reduce the number of trainable weight parameters. The optimal number of channels can improve the classification performance of the SER model.

For this experiment, we individually trained and evaluated the eight different CNN-based models with variant channel sizes. The detailed number of channel sizes is set with the parameter *n*, as listed in Table 3. Parameter *n* was selected as an integer ranging from 1 to 8. In addition, we used eight different versions of datasets to analyze which version of the preprocessing methods best represents the emotional features. The preprocessing methods are listed in Table 1.

**Fig. 6:**
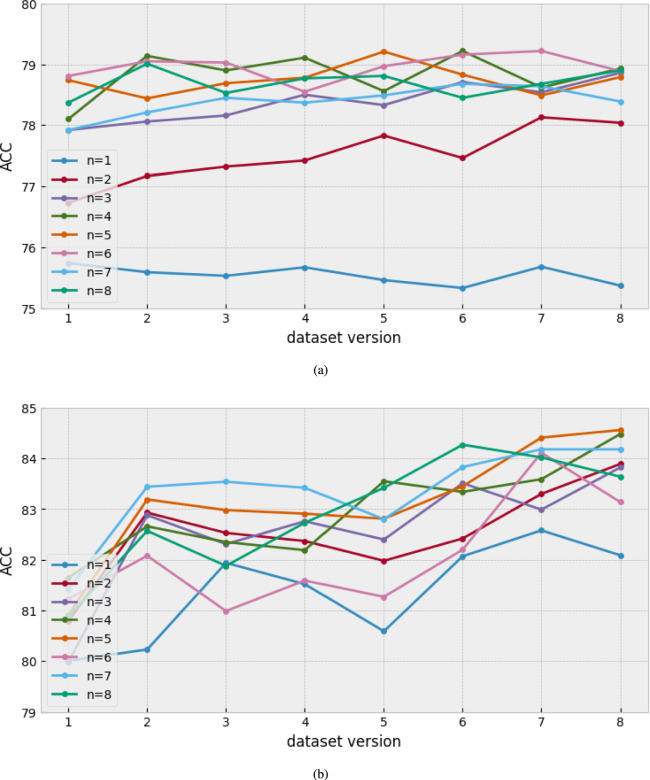
The comparison of ACC results from models using different channel sizes and versions of the dataset. (**a**) the result with IEMOCAP. (**b**) the result with EMODB.

Figure 6 shows the performance of the entire model for the different versions of datasets with IEMOCAP and EMODB corpus. With the perspective of channel size change, in Fig. 6a, the model performance changes drastically as the channel size increases in the range from 1 to 3. In particular, when comparing the performance of the $n=1$ (blue line) and $n=3$ (purple line) models, there was a $2 \% \sim 3 \%$ gap. Similarly, in Fig. 6b, model performance changes as the dataset version increases in the range from 1 to 2, has a $2\%$ gap. However, no improvement was observed in the models using a larger channel size ($(3\le n \le 8)$). It shows that a larger channel size can be inefficient for training the SER. Instead, an appropriate channel size can lead to a decent performance in emotion recognition.

Next, with the perspective of different versions of datasets, in Fig. 6a, except $n=1$, the best performance of each model can be observed in the higher versions of the datasets ($5 \sim 8$). Also, in Fig. 6b, it shows more concretely that the model performances increase with the higher versions of the datasets ($1 \sim 8$). This implies that a larger window size can effectively represent emotional features.

In summary, the channel size is an important factor in the performance of a CNN-based model. Also, enhancing the frequency resolution is more helpful for classifying the emotions. Therefore, in the next experiments, we explored how to improve the training channel features using the ECA with the effective STFT preprocessing datasets. To do so, we selected the models that achieve the best performance in SER. (IEMOCAP: $n=4$ (khaki line) with the dataset of version 6 (79.16 UA 79.27 WA 79.22 ACC), EMODB: $n=5$ (orange line) model with the dataset of version 8 (84.01 UA 85.05 WA 84.52 ACC))

### Using original ECA method in CNN-based models

To efficiently increase the channel feature representation, we use the ECA blocks in a CNN-based model. To do so, we first experimented with the original ECA block in selecting the CNN-based model (IEMOCAP: $n=4$, EMODB: $n=5$) that achieved the best performance in a channel size search experiment in the before subsection. To follow the original ECA adaptation manner, the original ECA is positioned after all convolution layers. Specifically, six ECA blocks were added to the CNN-based model. Next, the ECA’s kernel sizes (*k*) were set with respect to the channel size of each layer.

**Fig. 7:**
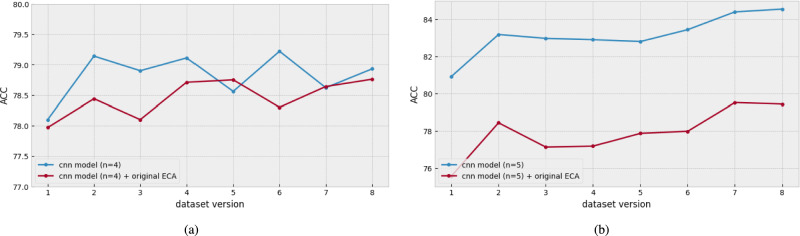
The comparison of ACC results of the original ECA block used or not in the CNN-based model. (**a**) the result with IEMOCAP. (**b**) the result with EMODB.

Figure 7 shows the performance of each dataset before and after applying the original ECA. Compared with the CNN-based model, the original ECA method (red line) showed an overall decrease in emotion recognition performance. In Fig. 7a, the performance decreased from $0.1\%$ to $1\%$. In Fig. 7b, the performance decreased from $2\%$ to $4\%$. This implies that the original ECA method is not suitable for direct application in the CNN-based model for SER. Therefore, we conducted experiments to determine a more effective way to use the ECA blocks in the CNN-based model for SER.

### Searching the proper ECA block usage in CNN-based models

To effectively use ECA blocks, we experimented with how the model performance changes when using the ECA blocks in different positions on a CNN-based model with different kernel sizes (*k*) and versions of the dataset. The CNN-based models were selected based on the results of Fig. 6 (IEMOCAP: $n = 4$, EMODB: $n = 5$).

A CNN-based model has a structure in which the channel size increases as the layer deepens; therefore, the complexity of the channel features in deep layers is relatively high. Based on that situation, we want to determine where the ECA block can be more effective in helping to train the channel features.

First, we examined the effect of the ECA block using eight different emotional speech preprocessing datasets. For this purpose, a CNN-based model with the ECA blocks on the fifth and sixth convolution layers was used. In addition, we changed the kernel size of the 1-d convolution layer of the ECA block with four kernel sizes (3, 5, 7, and 9) to verify the change in performance according to the kernel size. Moreover, we excluded the cases in which the kernel size was larger than nine because of poor performance.

**Fig. 8:**
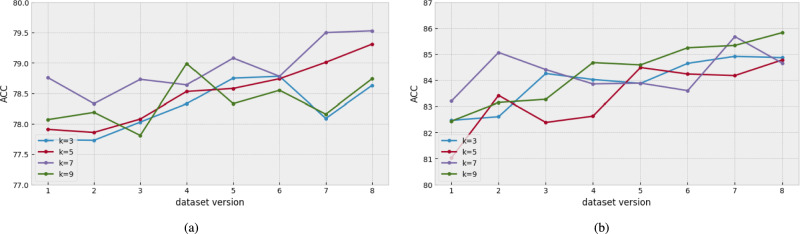
The comparison of ACC results used different versions of datasets with the CNN-based model using the ECA blocks in layers 5 and 6. (**a**) the result with IEMOCAP. (**b**) the result with EMODB.

As shown in Fig. 8, the model performance tended to increase from dataset versions 1 to 8. In particular, the version 8 dataset showed better results than the other version datasets in most cases. This indicates that a large-sized window in emotional speech preprocessing is effective when we use the ECA block. And, the best kernel size is increased to treat the large number of channel features. Specifically, in Fig. 8a, kernel size $(k = 7)$ shows the best performance around all versions of the dataset, except version 4. Also, in Fig. 8b, kernel size $(k = 9)$ shows the best performance in most datasets. It means that kernel size selection is also important to use the ECA block properly.

**Fig. 9:**
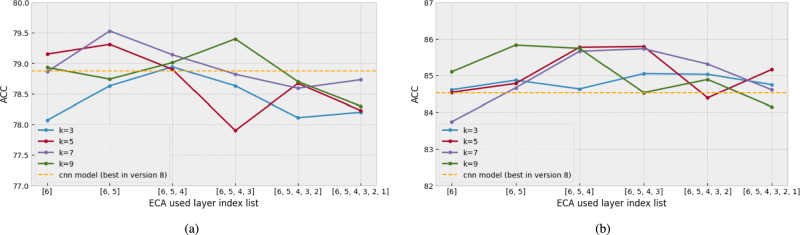
The comparison of ACC results with the ECA blocks used in different layers of the CNN-based model with different kernel sizes in the version 8 dataset. (**a**) the result with IEMOCAP. (**b**) the result with EMODB.

For that reason, with dataset version 8, we search more deeply into how to use the ECA block. Specifically, the experiment was conducted by sequentially adding the ECA blocks starting from the sixth convolution layer, which was the deepest layer in the model. And we also use four kernel sizes (3, 5, 7, and 9). The result can be found in Fig. 9.

First, if the results are analyzed according to the position of ECA blocks, Fig. 9ashows that the performance decreases when ECA blocks are used after most convolution layers, whereas the performance improves when ECA blocks are used for relatively deep layers (3 to 6 layers). Also, in Fig. 9b, the performance when ECA blocks are used for relatively deep layers is higher than others. These results show that the ECA block positioned in the deep-layer channel features was effective.

Second, if the results are analyzed according to the kernel size, in Fig. 9a, the best performance is $k = 7$ (purple line). Compared with the other kernel sizes (3, 5, and 9), $k = 7$ had an overall high accuracy range ($78.59 \sim 79.53$ ACC). However, $k = 7$ did not always exhibit the best performance in any of the cases. For example, the four ECA blocks used in model $k = 9$ (khaki line) performed better than the others (3, 5, and 7). This tendency is similar in Fig. 9b. The best performance model in Fig. 9bis in $k = 9$ (green line). However, the performance degraded when the ECA block was used with more than 4 CNN layers. It implies that the position of the ECA block in the deep-layer is much more important than the kernel size.

Consequently, with IEMOCAP, the proposed ECA method obtained a $1\%$ higher performance than that without an ECA block (dataset version 8, 79.37 UA 79.68 WA 79.53 ACC). Also, with EMODB, the proposed ECA method obtained a $1.5\%$ higher performance than before (dataset version 8, 85.48 UA 86.17 WA 85.83 ACC). This result indicates that the channel features of deeper layers are particularly important for extracting the speech emotion features. With our ECA block, increasing only a small number of trainable parameters in the CNN-based model can efficiently improve the speech emotion classification performance.

### Augmentation method with different versions of STFT datasets

To overcome the limitations of representing speech emotional features obtained using only one preprocessing method, multiple preprocessing data augmentation experiments were performed. For this purpose, only the training dataset was added from the eight different versions of the datasets obtained by the setting listed in Table 3. Because each of the eight preprocessing methods has a different window size and overlap size to complement the time-frequency resolution, the model can be trained with richer emotional features.

We conducted two different experiments depending on the dataset selection methods to determine the effect of multiple preprocessing data augmentation on SER. In the first case, we selected version 1 as the test set and collected training data samples from version 1 to version 8 in ascending order. Second, in contrast to the first, we selected dataset version 8 as the test set and collected the training data samples in descending order from version 8 to version 1. The models used in the experiment are CNN-based models with ECA blocks and models without an ECA block.

**Fig. 10:**
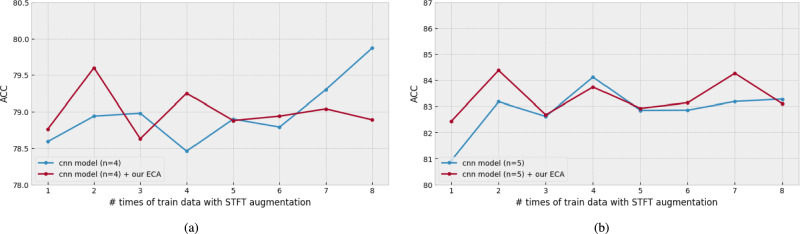
The comparison of ACC results used the STFT data augmentations. The number of the dataset is selected with the dataset versions in ascending order. (**a**) the result with IEMOCAP. (**b**) the result with EMODB.

Figure 10 shows the augmentation experiments in ascending order. In most cases, the results were higher than those in the cases where the augmentation method was not applied to either model. Specifically, in Fig. 10a, the best results in the CNN-based model were obtained using all the preprocessing datasets (80.10 UA 80.02 WA 80.06 ACC). In the case of models with ECA blocks, the best results were obtained when versions 1 and 2 were used (79.69 UA 79.51 WA 79.60 ACC). And, in Fig. 10b, the best results in the CNN-based model were obtained using versions 1 to 4 were used (83.75 UA 84.49 WA 84.12 ACC). In the case of models with ECA blocks, the best results were obtained when versions 1 to 2 were used (84.28 UA 84.49 WA 84.38 ACC). These results show that the multiple preprocessing augmentation method can improve performance when a small amount of data is available.

**Fig. 11:**
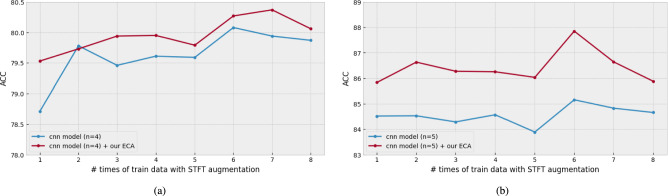
The comparison of ACC results used the STFT data augmentations. The number of the dataset is selected with the dataset versions in descending order.(**a**) the result with IEMOCAP. (**b**) the result with EMODB.

Next, Fig. 11 shows the augmentation experiments in descending order. An interesting result shown in Fig. 11a is that the CNN-based model (79.66 UA 80.50 WA 80.08 ACC) and the CNN-based model with ECA blocks (80.28 UA 80.46 WA 80.37 ACC) exhibited the highest performance. Compared with the ascending experiment, the CNN-based model with ECA blocks showed $0.8\%$ higher. In addition, the CNN-based model with ECA blocks (red line) is usually $0.2\%$ to $0.82\%$ higher than that of the CNN model (blue line). In other words, the multiple preprocessing augmentation method can significantly improve the learning of emotional features using ECA blocks.

Moreover, in Fig. 11b, the CNN-based model with ECA blocks achieves the highest performance (87.67 UA 88.04 WA 87.85 ACC). Compared with the models that did not use STFT augmentation, the performance was improved by almost $2\%$ when versions 8 to 3 were used. From these two experiments, we can observe that the multiple preprocessing augmentation method not only compensates for the training problem with a few speech-emotional data samples but also enhances the time-frequency resolution, which is one of the difficulties in SER. In addition, the ECA blocks can work more effectively using the data augmentation method.

### Comparison with other attention models

**Table 4:** The comparison of performance with different SER models in IEMOCAP dataset.

Model	Method	Year	UA	WA	ACC	Total Parm	FLOPs
Head-fusion^54^	multi-head attention	2020	70.1	76.4	73.3	614,180	34.32 G
HNSD^14^	LSTM-GMU-Attention	2021	72.5	70.5	71.5	3,078,988	0.136 G
Area Attention^31^	Multi-scale area attention	2021	77.5	79.3	78.4	156,156	7.84 G
ATDA^32^	Multi-view, granularity	2022	75.4	76.2	75.8	630,458	16.44 G
STC^33^	STC attention	2023	61.8	61.8	51.8	112,894,764	15.50 G
AMSNet^34^	connection attention	2023	70.5	69.2	69.9	11,159,376	0.0286 G
Vesper^59^	transformer	2024	73.9	73.0	73.45	63,515,872	35.16G
Our method	ECA, STFT augmentation		**80.3**	**80.5**	**80.4**	2,734,482	61.10 G

**Table 5:** The comparison of performance with different CNN-based attention.

CNN-based attention	Parameter complexity	IEMOCAP			EMODB		
		UA	WA	ACC	UA	WA	ACC
Head-fusion^54^	$O(C*d_{head})$	78.59	78.86	78.73	84.22	84.67	84.45
Area Attention^31^	$O(C^2)$	74.85	75.60	75.09	84.45	84.67	84.56
STC^33^	$O(C^2 + K)$	79.30	78.90	79.10	84.89	85.79	85.34
MHA^60^	$O(C^2)$	73.96	73.63	73.79	80.87	81.12	81.00
ECA	*O*(*K*)	**79.37**	**79.68**	**79.53**	**85.48**	**86.17**	**85.83**

**Table 6:** The results of ablation.

Method	Loss	IEMOCAP			EMODB		
		UA	WA	ACC	UA	WA	ACC
CNN-based model	cross entropy	78.62	78.70	78.66	83.54	84.67	84.11
	weighted focal	78.94	78.80	78.87	84.01	85.05	84.52
+ Original ECA block	-	78.71	78.80	78.76	78.86	80.19	79.44
+ Our ECA block	-	79.37	79.68	79.53	85.48	86.17	85.83
+ STFT augmentation	-	79.66	**80.50**	80.08	84.71	85.61	85.16
+ + Our ECA block	-	**80.28**	80.46	**80.37**	**87.67**	**88.04**	**87.85**

We compare the ECA CNN-based model with other proposed models that use attention methods. For this, we chose our best results models that contained ECA blocks and STFT data augmentation. Table 4 lists the results of the UA, WA, and ACC evaluations. Also, for a clearer comparison of model specifications, we added the number of learning parameters and FLOPs used in each model. All selected models used attention methods and were evaluated with the 5-fold cross-validation using the IEMOCAP dataset.

As listed in Table 4, the proposed model shows a significantly better balanced performance than the other models. This is because the combination of deep CNN layers and ECA is an effective structure for extracting emotional context. In addition, the insufficient expression of emotional features can be reinforced using the STFT augmentation method. Therefore, for the SER, it is important to increase the representation of the emotional feature data and effectively learn the context within it.

Next, to show the effectiveness of the ECA module in the CNN-based model, we adopted different types of CNN-based attention methods in our CNN-based model. Table 5 lists the parameter complexity of each CNN-based attention method and the results of the UA, WA, and ACC evaluations with IEMOCAP and EMODB datasets. Because the ECA’s parameter complexity isn’t dependent on the number of filters (*C*) in CNN, it has high efficiency in parameter complexity. Moreover, compared to the other attention method, with the ECA module, the result was recorded as the highest score. In summary, the ECA module shows meaningful performance with a small number of parameters *O*(*k*).

### Analysis of the ablation studies

For a detailed analysis of the proposed methods, the results of the ablation models were compared. With dataset version 8, Table 6 lists performance depending on whether or not the ECA block and STFT data augmentation were used. From the overall results, we can observe that the emotion classification performance is improved when applying our proposed ECA block. In particular, the effect of the ECA block can significantly improve performance when used together with the STFT data augmentation method.

**Fig. 12:**
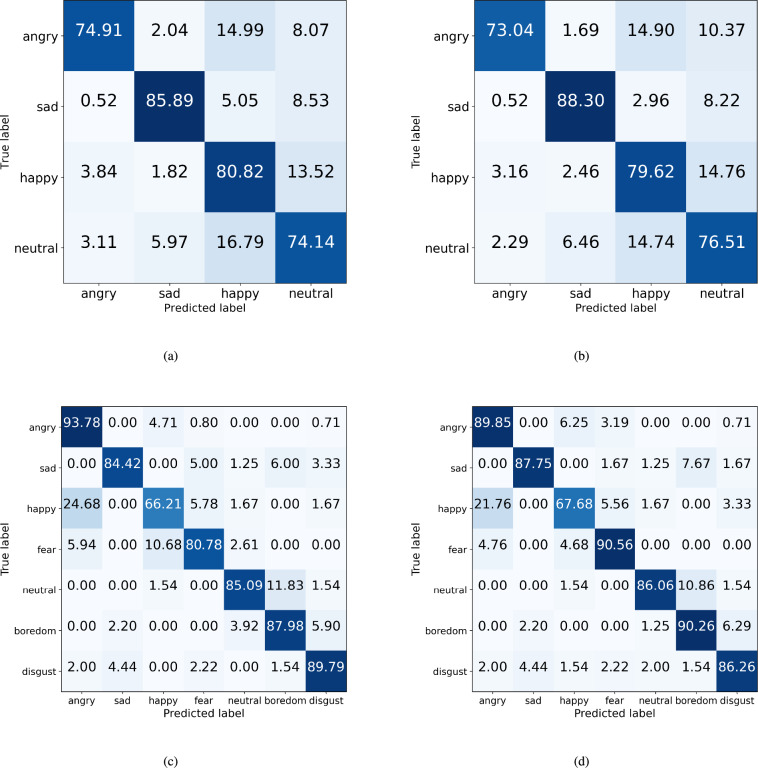
The confusion matrices whether the ECA block is used or not in the CNN-based model. (**a**) CNN-based model ($n=4$) with IEMOCAP (**b**) ECA block used in layers 5 and 6 with IEMOCAP (**c**) CNN-based model ($n=5$) with EMODB (**d**) ECA block used in layers 5 and 6 with EMODB.

**Fig. 13:**
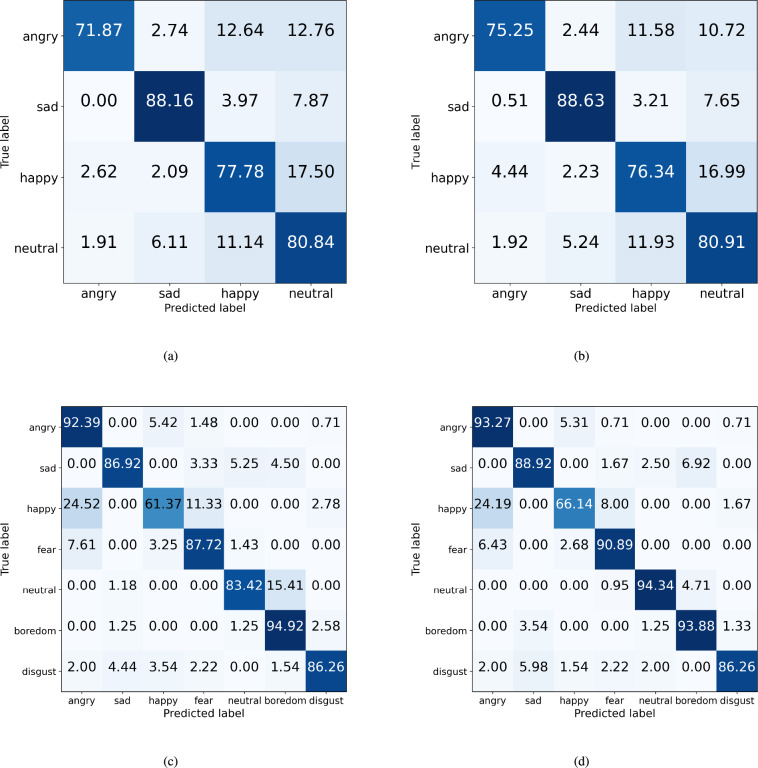
The confusion matrices whether the ECA block is used or not in the CNN-based model with STFT augmentation method. (**a**) CNN-based model ($n=4$) with IEMOCAP (**b**) ECA block used in layers 5 and 6, with IEMOCAP (**c**) CNN-based model ($n=5$) with EMODB (**d**) ECA block used in layers 5 and 6 with EMODB.

Subsequently, we compared the classification performance for each emotion according to the ablation models. Figures 12 and 13 show the confusion matrices of the ablation models with the IEMOCAP and EMODB datasets. With the IEMOCAP dataset, in most models, the classification performance of sadness was high, and the classification performance for anger, happiness, and neutral tended to be low. However, compared with Fig. 12a, Fig. 12b shows that most of the classification performance of emotions improved when the ECA and STFT augmentation methods were used.

Specifically, in Fig. 12a and b, the sadness classification accuracy improved by 2.5%, and the neutral classification accuracy decreased by 2.4% using the ECA. It means that the ambiguity of classifying sadness and neutral was resolved through the ECA block. Next, in Fig. 13a and b, the neutral classification accuracy improved by approximately 2% $\sim$ 4%. In particular, in Fig. 13b, both angry and neutral classification accuracies were significantly improved.

Next, with the EMODB dataset, in Fig. 12c, the emotions of anger, happiness, fear, neutral, and boredom are more highly confused than others. However, when the ECA and STFT augmentation methods were used, this incorrectness was resolved. In particular, in Fig. 13d, the incorrectness between neutral and boredom was notably decreased by 6% $\sim$ 11% compared with other models. Also, the classification accuracy of anger, fear, neutral, and boredom is over 90%. In summary, the proposed method can increase the most of accuracy of emotion classification.

### Analysis of the channel scores of ECA module

**Fig. 14:**
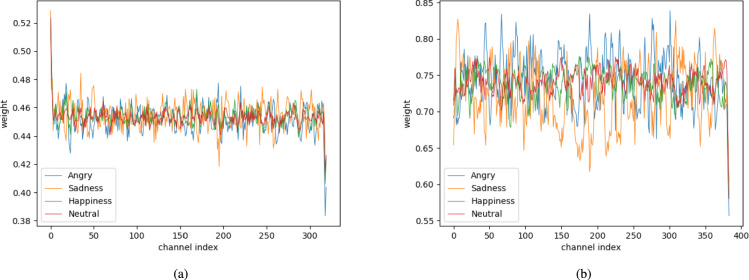
The ECA’s channel weight plots of each emotion class with IEMOCAP. (**a**) The channel weights in layer 5. (**b**) The channel weights in layer 6.

**Fig. 15:**
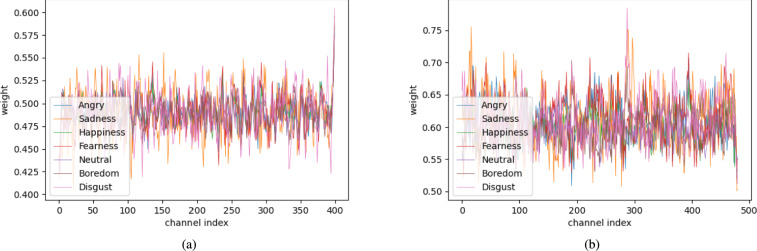
The ECA’s channel weight plots of each emotion class with EMODB. (**a**) The channel weights in layer 5. (**b**) The channel weights in layer 6.

The results show that channel feature extraction with the ECA blocks is effective for SER. Subsequently, to understand how the ECA block works for classifying each emotion, we checked the channel weights for each emotion. Figures 14 and 15 show a plot of the channel weight of the ECA blocks learned in the 5th and 6th layers when using the STFT augmentation method. To plot the channel weights, the weights from the test dataset were averaged.

As shown in Fig. 14a and Fig. 14b, the ECA weights of the fifth layer are slightly different between emotions. However, despite these channel weights having less variance than ECA weights of the last layer, sadness (orange line) weights were distinguishable from other emotions’ weights with the highest accuracy. It means that the channel weights can imply the original emotional features.

Next, in Fig. 15a and b, the ECA weights of the sixth layer are noticeably different. These channel weights have more variance than the ECA weights of the fifth layer. This phenomenon leads the model can easily classify the emotions. Specifically, in Fig. 15a, channel weights were distinguishable between angry (blue line) and sadness (orange line). In addition, neutral (red line) is distinct from angry (blue line) and sadness (orange line). However, it is slightly different from the neutral (red line) to happiness (green line), because it is difficult to distinguish between them. In summary, adopting the ECA module in the CNN-based model can effectively help to train the emotional context more distinguishably.

## Discussion

Our experimental results show that the enhancement of the channel feature is closely related to performance in training the CNN model for SER. In addition, ECA elaborates channel feature representation more efficiently than any other attention method, thereby improving its performance. In particular, ECA plays a much more important role when learning with wide feature spectrum speech data by STFT augmentation. Therefore, an improved attention module that is more suitable for CNN is an important element in the SER.

However, our experiment has some limitations. First, since we empirically investigated the effect of channel filters on emotion recognition problems, it is difficult to establish a theoretical basis for why a specific number of channel filters is more effective or better. This is because it is difficult to mathematically connect the channel filters with the data size and the number of classification labels in other problems that mainly use CNN. However, as some empirical studies have shown, the number of channel filters that need to be used increases as the complexity of the data set increases or the amount of data increases^61^. However, the SER problem has relatively less data than other problems. Therefore, it is necessary to study how to have a clear role for each filter with an appropriate number of channel filters^62^.

The second limitation is that ECA only considers local interactions when distinguishing between channel filters. This means that ECA cannot learn the relationship of the entire channel filter, which makes it difficult to consider the features of a more comprehensive speech signal. However, ECA is also strong in problems such as image classification that require classification of many types^45^. Therefore, it can work well in tasks such as SER, which classifies a small number of emotions rather than classifying various emotions. As shown in Table 5 through experiments, we showed that ECA can be more effective than other attention methods in the SER problem because it can focus on the local features of each emotion.

Finally, we would like to discuss the limitations of deploying the model in real-world scenarios. Since the SER model uses personal speech data as input, sensitive measures are required to protect users’ data privacy^63^. One approach to address this is to configure encrypted voice data along with CNN-based models within a Machine Learning as a Service (MLaaS) framework. In particular, methods tailored to CNN model architectures that utilize Homomorphic Encryption (HE)^64^ are efficient and tend to incur less performance degradation, making them suitable for real-world applications. HE enables an end-to-end process that encrypts both the original data and the model, thereby reducing the risk of exposing personal emotional characteristics throughout the entire pipeline^65–67^. Furthermore, since all the models we propose are CNN-based, including those with ECA, they are well-suited for such encryption techniques. Another privacy-preserving alternative is to enhance personal privacy by generating new emotional voice data using generative adversarial network (GAN)-based models^68^. This approach not only alleviates the issue of insufficient GAN-based emotional speech data but also contributes to privacy preservation^69^.

## Conclusion

This study proposes a promising and more effective preprocessing method and an ECA module for SER, offering the potential for significant advancements in the field. Our experiment, conducted with eight different preprocessing datasets from the IEMOCAP corpus and the EMODB dataset, revealed a significant finding: a spectrogram with a higher frequency resolution is more effective in training emotional features, providing valuable insight for future research in the field. Our study is the first study to apply an ECA to the SER. We achieved significantly better results than previous models by applying ECA to our CNN-based model with an effective preprocessing method.

Considering these results, correctly understanding the relationship between the channel features in the CNN structure can be a clue to understanding how to distinguish emotions. However, the ECA is limited in that it only considers the relationship between neighboring channels. In future work, we will look for attention structures that are efficient but can learn the relationships between channel features more broadly. In addition, it is necessary to determine a better preprocessing method for emotion recognition by analyzing the frequency features associated with each emotion.

## Data Availability

The interactive emotional dyadic motion capture (IEMOCAP) corpus and Berlin Emotional Speech Database (EMODB) are available at the websites (IEMOCAP: https://sail.usc.edu/iemocap, EMODB: http://emodb.bilderbar.info/start.html)
